# Biochemical urine analysis of atorvastatin and rosuvastatin by LC-MS: a pilot study of an objective method to assess non-adherence

**DOI:** 10.1186/s12872-025-05475-0

**Published:** 2026-01-09

**Authors:** Maximilian Brockmeyer, Nadja Chernyak, Daniel Dröge, Hanna Wessel, Claudio Parco, Alexander Hoss, Kris G. Vargas, Yingfeng Lin, Annette Rickert, Stefanie Ritz, Andrea Icks, Malte Kelm, Georg Wolff, Oliver Temme

**Affiliations:** 1 Department of Internal Medicine, Division of Cardiology, Pulmonology and Vascular Medicine, Medical Faculty and University Hospital Düsseldorf, Heinrich Heine University Düsseldorf, Moorenstr. 5, Düsseldorf, Germany; 2 Institute for Health Services Research and Health Economics, Centre for Health and Society, Medical Faculty and University Hospital Düsseldorf, Heinrich Heine University Düsseldorf, Düsseldorf, Germany; 3 Institute for Health Services Research and Health Economics, German Diabetes Center, Leibniz Center for Diabetes Research at the Heinrich Heine University Düsseldorf, Düsseldorf, Germany; 4 Clinic for Cardiology, Klinikum Ibbenbüren, Ibbenbüren, Germany; 5 Nuffield Department of Population Health, University of Oxford, Oxford, United Kingdom; 6 Department of Forensic Toxicology, Institute of Forensic Medicine, Medical Faculty and University Hospital Düsseldorf, Heinrich Heine University Düsseldorf, Düsseldorf, Germany; 7 CARID – Cardiovascular Research Institute, Düsseldorf, Germany

**Keywords:** Atorvastatin, Rosuvastatin, Urine, Liquid chromatography/Mass spectrometry, Non-adherence, Coronary artery disease

## Abstract

**Objective:**

Non-adherence to statins represents a contributing factor for poor attainment of low-density lipoprotein cholesterol treatment goals in secondary prevention of coronary artery disease (CAD). We aimed to establish urine analysis by liquid chromatography/mass spectrometry (LC-MS) as a method for objective measurement of non-adherence to high-potency statins. Additionally, we sought to apply the method to a population of ambulatory CAD patients at a German heart center in a cross-sectional pilot study.

**Methods:**

Volunteers provided urine samples one, two, three, and seven days after intake of a single dose of atorvastatin and/or rosuvastatin. Additionally, urine samples from ambulatory CAD patients on prescription of atorvastatin or rosuvastatin were obtained. All urine samples were analyzed by LC-MS for concentrations of atorvastatin or rosuvastatin. Lower limits of detection were determined on spiked urine samples to define cut-off values (COVs) for the detection of statins (atorvastatin 1.40 ng/mL, rosuvastatin 1.00 ng/mL). Non-adherence was defined as a measurement of the respective statin below the COV.

**Results:**

On day one, in volunteers’ (*n* = 16, 37.3 ± 6 years, 81.3% male) urine samples, concentrations were above COV in 92.3% (atorvastatin, *n* = 13) and 90% (rosuvastatin, *n* = 10). After seven days, atorvastatin could not be detected in all 13 volunteers (100%) and rosuvastatin in nine of ten volunteers (90.0%),

**Conclusion:**

LC-MS analysis of urine is a feasible method for direct testing of adherence to high-potency statins within a one-week time-window. In the investigated population of ambulatory patients with CAD, non-adherence to statins was found in a considerably low range.

**Trial registration:**

ClinicalTrials.gov ID NCT05814692, first registered 20,230,403.

**Supplementary Information:**

The online version contains supplementary material available at 10.1186/s12872-025-05475-0.

## Introduction

Low-density lipoprotein cholesterol (LDL-C) is considered a causal factor for development of atherosclerotic cardiovascular disease (ASCVD) [1], including coronary artery disease (CAD), which is the leading cause of death worldwide [2]. Hence, lowering LDL-C is of paramount medical, social, and economic importance in the prevention of ASCVD [3]. As recommended by international cardiovascular guidelines, high-potency statins (i.e., atorvastatin and rosuvastatin) represent a cornerstone of current lipid-lowering medication to achieve LDL-C treatment goals [4]. However, data from large international registries show poor attainment of these treatment goals in highest cardiovascular risk patients, with a great need for improvement [5, 6]. Data from our institution also showed that ASCVD patients attain LDL-C treatment goals infrequently [7, 8]. 

Successful secondary prevention depends on good patient adherence to prescribed pharmacotherapy. Unfortunately, adherence to statin-therapy is particular non-satisfactory as compared to other cardiovascular drugs [9]. There is evidence that adherence to statin therapy decreases by approximately 50–60% within one year of initiation in some populations [10]. For developing successful interventions aiming to improve adherence to statin therapy, precise measurement of adherence is mandatory, which is challenging. In many studies, measurement of adherence relied on patient self-reports that may be prone to bias [11]. Biochemical analysis of urine is a promising objective method to measure adherence [12]. 

Within the present work, we aimed to establish urine analysis by liquid chromatography/mass spectrometry (LC-MS) as a method for objective measurement of non-adherence to high-potency statins. In a second step of this pilot study, we then applied the method to a population of ambulatory CAD patients at our German heart center.

## Materials and methods

### Study design

This cross-sectional pilot study consisted of two phases:

First, to establish analytical methods of LC-MS and to gain insights into the time course of urine excretion of high-potency statins measured by LC-MS; thus, healthy volunteers received a single dose of atorvastatin or rosuvastatin and were investigated by serial urine testing up to one week after administration of statins.

Second, to evaluate the method in a real-world setting; thus, ambulatory patients with CAD on atorvastatin and rosuvastatin were tested by single spot urine LC-MS analysis.

The protocol of the study has been positively evaluated by the ethics committee of the Medical Faculty of Heinrich-Heine-University Düsseldorf (Study-ID 2022 − 1927).

### Subjects

#### Volunteers

Healthy volunteers, who were not on previous lipid-lowering medication, agreed to participate: All were medical doctors, who gave consent to self-experiment after a detailed conversation about purposes of the study and potential risks.

#### Patients with CAD

Outpatients ≥ 18 years of age with known CAD, who were attending a consultation at our tertiary care German heart center in Düsseldorf and were on guideline-recommended atorvastatin or rosuvastatin on regular prescription, were eligible for inclusion after written informed consent.

### Collection of urine samples

#### Volunteers

All volunteers received a single dose of 40 mg atorvastatin or 20 mg rosuvastatin as a commercially available pill between 06:00 and 08:00 p.m. The first urine sample was taken between 06:00 and 10:00 a.m. on the following day (day 1, ~ 12 h after intake). Additional urine samples were taken between 06:00 and 10:00 a.m. on day 2, day 3, and day 7 after application of the statin dose. All urine samples were immediately packaged in light-proof envelopes and stored at -18 °C, to be transferred on ice to the laboratory of the department of forensic toxicology for storage at -18 °C until further analysis.

#### Patients with CAD

Patients provided a urine sample, which was immediately packaged in light-proof envelopes and stored at -18 °C within the outpatient department. After all patient urine samples of a day were collected, they were again transferred on ice to the laboratory of the department of forensic toxicology for storage at -18 °C until further analysis. Patients were not aware of the collection of a urine sample prior to study inclusion, which took place at the same time of their scheduled appointment in the outpatient department.

### Sample Preparation and LC-MS parameters

15 µl of the deuterated substances (d5-atorvastatin and d3-rosuvastatin, concentrations each 2 ng/µL, TargetMol Chemicals Inc., Boston, USA) were added to 100 µL of a urine sample, followed by 150 µL of an acetonitrile/water solution (50/50 v/v, LC-MS grade, VWR chemicals of Avantor Inc., Radnor, USA). After mixing and centrifugation, 100 µL were transferred to an autosampler vial. From this, 10 µL were injected into the LC-MS system.

An ACQUITY ultra performance (UP) LC system (Waters Corp., Milford, USA) with an ACQUITY TQD triple quadrupole tandem mass spectrometer (MS/MS) with electrospray ionization (Waters Corp., Milford, USA) was used. Chromatography was performed using a ACQUITY UPLC HSS C18 column (1,8 μm x 2,1 mm x 150 mm, Waters Corp., Milford, USA). For mobile phase A, ammonium formiate in water (5 mmol/L, pH 3, LC-MS grade, VWR chemicals of Avantor Inc., Radnor, USA) was used. Mobile phase B consisted of acetonitrile (LC-MS grade, VWR chemicals of Avantor Inc., Radnor, USA). Detailed parameters of the UPLC ACQUITY system selected for the analyses can be.

found in Table 1.

For reasons of internal quality control, to account for possible contaminations from previous samples, standard blank urine (from the laboratory’s sample inventory, i.e., known not to be contaminated with statins) was injected and tested in the LC-MS systems before each measurement of volunteer and patient urine.

**Table 1:** LC-MS parameters

Chromatographic condition	Value	
Column	ACQUITY UPLC HSS C18 (1.8 μm x 2.1 mm x 150 mm, Waters Corp., Milford, USA)	
Column temperature	50 °C	
Ambient temperature	10 °C	
Flow rate	0.45 mL/min	
Mobile Phase A	5 mM ammonium formiate in water (pH 3)	
Mobile Phase B	Acetonitrile	
Injection volume	10 µL	
Analysis time	11.5 min	
Gradient/time	Mobile Phase A	Mobile Phase B
Initial	87.0%	13.0%
0.5 min	87.0%	13.0%
9.0 min	5.0%	95.0%
10.0 min	5.0%	95.0%
12.5 min	87.0%	13.0%

Table 1: Parameters of liquid chromatography/mass spectrometry (LC-MS) for biochemical urine testing for atorvastatin and rosuvastatin. An ACQUITY ultra performance (UP) LC system (Waters, Milford, USA) with an ACQUITY TQD quadruple tandem mass spectrometer with electrospray ionization and column oven (MS/MS, Waters Corp., Milford, USA) was used.

### Cut-off value, validation, and definition of non-adherence

The limits of detection (LODs) and lower limits of quantification (LOQs) for atorvastatin and rosuvastatin were determined on spiked blank urine samples (TargetMol Chemicals Inc., Boston, USA) as described by German Institute for Standardization (DIN) standard 32,645: [13] LODs of atorvastatin and of rosuvastatin were 1.37 ng/mL and 0.93 ng/mL, LOQs 1.97 ng/mL and 9.07 ng/mL. Calibration by linear regression analysis was performed in the range of 2.5 to 250 ng/mL.

A partial validation was carried out as described by DIN standard 32,645 [13]. To asses selectivity, six blank matrix samples (standard blank urine) without deuterated standard substances along with two blank matrix samples with deuterated standard substances were analyzed: No interfering signals were detected. Stability was assessed over the time period of a sample sequence (0–36 h) by measurement of five spiked control samples for each test substance (i.e., atorvastatin and rosuvastatin) at high and low concentrations (20 ng/mL and 140 ng/mL). For both substances, instabilities were found in a low range at low (atorvastatin 12%, rosuvastatin 9%) and high concentrations (atorvastatin 7%, rosuvastatin 6%). Long-term stability was evaluated after storage at -18 °C for 175 days. Measurements of four spiked control samples each at low and high concentrations (20 ng/mL and 140 ng/mL) for atorvastatin and rosuvastatin showed results within the range of 90–110% of the actual concentration (atorvastatin + 3.8% and 3.7%, rosuvastatin + 3.0% and − 5.6%). Matrix effects were assessed by analyzing five spiked control samples compared to a spiked aqueous solution (LC-MS grade, VWR chemicals of Avantor Inc., Radnor, USA) at low (5 ng/mL) and higher (50 ng/mL) statin concentrations and calculated as a percentage of absolute areas (spiked urine/spiked aqueous solution). The observed matrix effects were negligible (atorvastatin 96% and 104%, rosuvastatin 97% and 106%).

The cut-off values for non-adherence derived from LC-MS analyses were defined qualitatively based on LODs. Cut-off values were rounded from LODs: For atorvastatin cut-off was set < 1.40 ng/mL, for rosuvastatin < 1.00 ng/mL. Patients with urine concentrations below these cut-offs measured by LC-MS were considered as non-adherent to high-potency statins.

### Statistics

Continuous data are presented as means ± standard deviations. Ordinal/categorical data are presented as counts and percentages of total. Data analysis was performed using SPSS 23.0 (IBM Corp., Armonk, USA).

## Results

### Healthy volunteers, detection of high-potency Statins over time

Sixteen volunteers (13 male (81.3%); mean age 37.3 ± 6 years; Table 2) agreed to participate. A series of four urine samples (days 1, 2, 3, and 7) was obtained from 13 volunteers after the intake of atorvastatin (83.3% male, mean age 36.9 ± 5 years), from ten volunteers after the intake of rosuvastatin (80.0% male, 37.8 ± 7 years). One subject developed an illness during the test series and could not provide adequately stored urine samples, which is why all samples were excluded from the analysis. One atorvastatin volunteer repeated statin intake and urine sample collection due to uncertainty about LC-MS results (see next paragraph).

**Fig. 1:**
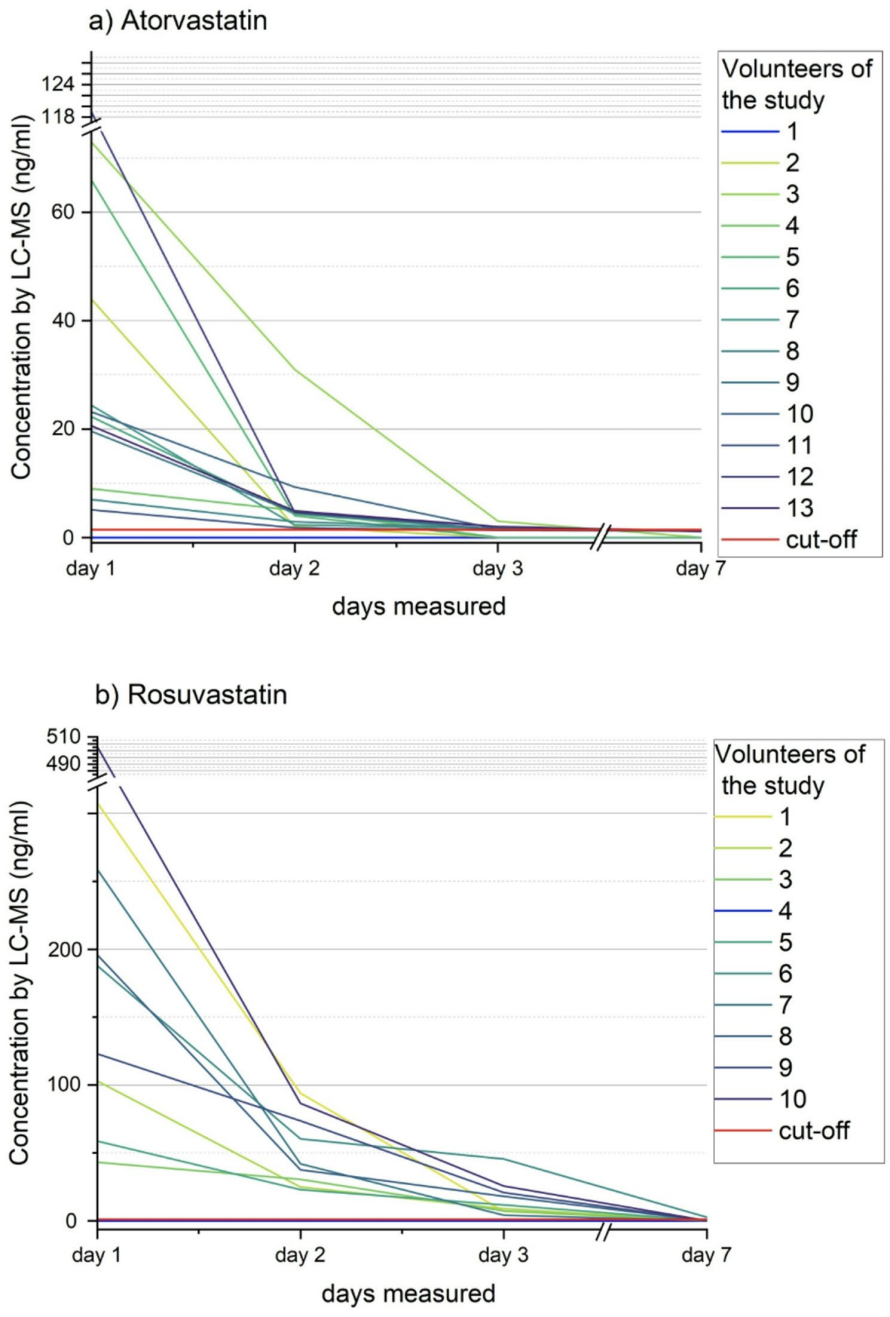
Detection of high-potency statins over time. Results of biochemical urine testing by liquid chromatography/mass spectrometry (LC-MS) in healthy volunteers one, two, three, and seven days after the intake of **(a)** atorvastatin (*n* = 13) and **(b)** rosuvastatin (*n* = 10)

Figure 1 shows individual results of LC-MS urine analysis of all subjects at all pre-defined timepoints (days 1 to 7). On day 1, urine analysis by LC-MS showed concentrations above the cut-off value for the analyzed high-potency statin in twelve of 13 urine samples after the intake of atorvastatin (92.3%), in nine of ten samples after the intake of rosuvastatin (90.0%). In urine samples from day 2, atorvastatin was detected in twelve subjects (92.3%), from day 3 in eight subjects (61.5%). Rosuvastatin was detected in nine of ten subjects on days 2 and 3 (90.0%). After one week (day 7), atorvastatin could not be detected in all 13 subjects (100%) and rosuvastatin in nine of ten subjects (90.0%). The urine samples of the first volunteer of the atorvastatin series showed concentrations below the cut-off value in all urine samples as analyzed by LC-MS. Since this was the first proband, the intake of atorvastatin was repeated and a second complete set of urine samples was provided and analyzed. Based on the experiences from analyzing atorvastatin concentrations with a proportion of false negative results of approximately 10.0%, the intake of rosuvastatin was not repeated in a volunteer with rosuvastatin concentrations below the cut-off value in all urine samples. The individual statin concentrations of the volunteers’ urine samples are listed in Supplementary Table 1. Additionally, exemplary chromatograms of the analysis urine samples of one volunteer are displayed in Supplementary Fig. 1.

Measurements of standard control urine that was injected into the LC-MS systems before each analysis of volunteer and patient urine for reasons of internal quality control did not produce any integrable peaks (i.e., no concentration was measurable).

### CAD patients, sample characteristics, and non-adherence

Table 2 shows characteristics of CAD patients: In total, 130 patients (81.5% male, mean age 65.6 ± 8 years) were included. Comorbidities associated with high cardiovascular risk were frequent (96.9% hypertension, 33.1% diabetes mellitus, 23.1% chronic kidney disease). The majority of patients (80.0%) was on prescription of atorvastatin. High-intensity statin therapy (i.e., ≥40 mg atorvastatin or ≥20 mg rosuvastatin per day) was prescribed in 78.5%.

**Table 2:** Study participants

Characteristics	Value
Volunteers (*n* = 16)	
Male	13 (81.3%)
Age (years)	37.3 ± 6
Statin intake	
Atorvastatin	13 (81.3%)
Rosuvastatin	10 (62.5%)
CAD patients (*n* = 130)	
Male	106 (81.5%)
Age (years)	65.6 ± 9
BMI (kg/m²)	28.5 ± 5
Hypertension	126 (96.9%)
Diabetes mellitus	43 (33.1%)
Chronic kidney disease	30 (23.1%)
eGFR < 30 mL/min/1,73m^2^	5 (3.8%)
Statin therapy	
Atorvastatin	104 (80.0%)
Rosuvastatin	26 (20.0%)
High-intensity statin therapy	102 (78.5%)

Table 2: Characteristics of study participants: Healthy volunteers (*n* = 16) with intake of a single dose of a high-potency statin (atorvastatin and/or rosuvastatin), coronary artery disease (CAD) patients (*n* = 130) on regular prescription of high-potency statin therapy. Data are presented as n (%) or as mean ± standard deviation. BMI = body mass index, eGFR = estimated glomerular filtration rate.

**Fig. 2:**
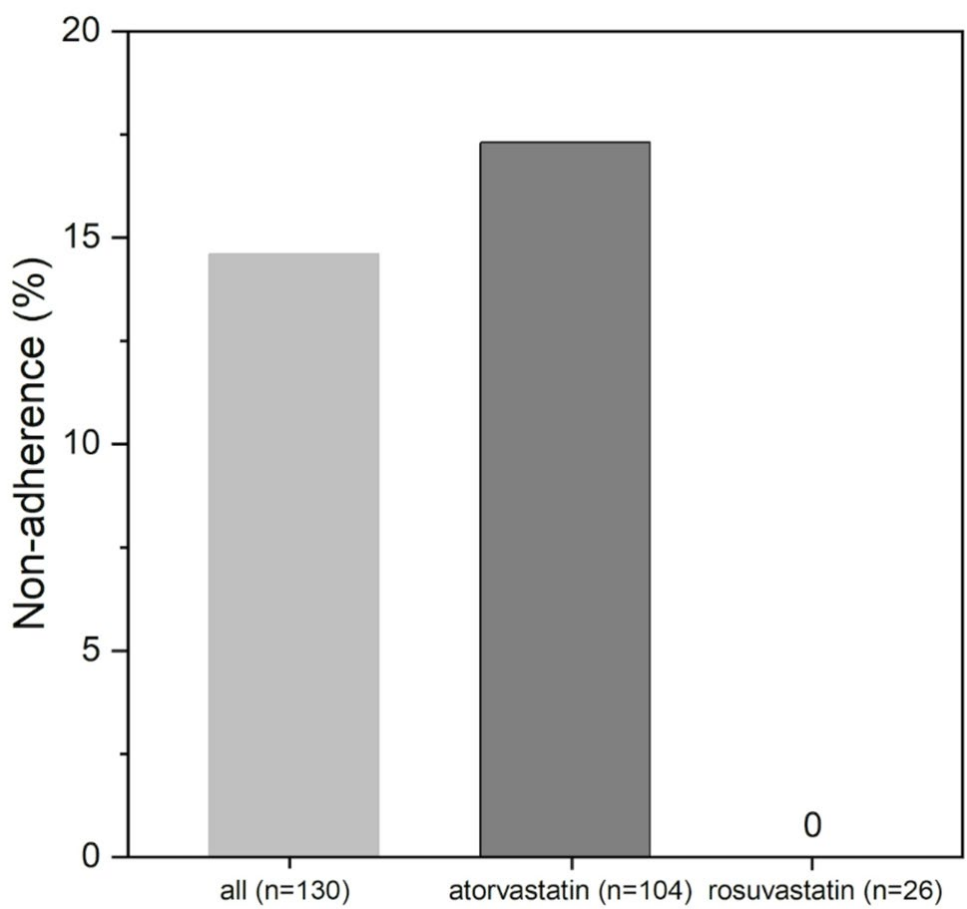
Non-adherence in ambulatory CAD patients measured by LC-MS. Results of analyzing non-adherence by biochemical urine testing by liquid chromatography/mass spectrometry (LC-MS) in ambulatory patients with coronary artery disease (CAD, *n* = 130) on prescription of atorvastatin (*n* = 104) or rosuvastatin (*n* = 26)

Overall, non-adherence to high-potency statin therapy was found in 13.8% of CAD patients (Fig. 2). Among patients on prescription of atorvastatin non-adherence was revealed in 17.3%, while none of the patients on prescription of rosuvastatin were classified as non-adherent (Fig. 2).

## Discussion

In this pilot study of an objective method to assess non-adherence to statins, we (a) established urine analysis of high-potency statins by LC-MS; (b) investigated diagnostic accuracy of the method; and (c) applied it to a population of ambulatory CAD patients to evaluate non-adherence in a real-world setting.

The main findings are as follows: (1) LC-MS analysis of urine samples is a feasible method for detecting non-adherence to high-potency statins for a time period of one week or longer; (2) non-adherence to high-potency statins was infrequent in the investigated CAD population; (3) non-adherence to atorvastatin was found more frequently as compared to rosuvastatin.

Among others, non-adherence to prescribed lipid-lowering statin therapy is a contributing factor to current non-satisfactory state of LDL-C risk factor control. LC-MS analysis of urine for objective measurement of adherence to cardiovascular pharmacotherapy might serve as a basis for the development of complex interventions to improve adherence [12]. 

The present experiences with healthy volunteers indicate that LC-MS methodology is a feasible method for assessment of non-adherence to high-potency statins of one week or longer providing reasonable diagnostic precision: For both atorvastatin and rosuvastatin false negative results were found in approximately one of ten patients (i.e., sensitivity estimated ≥90%) and a single dose was not detectable after one week in 90–100% of volunteers. Further efforts should be made to refine the methodology to reduce the rate of false negative results. A conceivable way of enhancing sensitivity would be to measure not only the parent drugs but also their active metabolites [14]. Otherwise, in unfavorable constellations involving specific patient groups, e.g., very small patient cohorts or those with low adherence, clinical decisions might be derived from incorrect assumptions regarding non-adherence.

With regards to cardiovascular drugs, biochemical urine testing of adherence by LC-MS was initially established in patients with hypertension and later extended to patients with diabetes, heart failure, and transient ischemic attack [15–18]. However, drug intake was not verified or tested in volunteers and therefore, information on diagnostic accuracy is lacking from these studies [15–18]. Previous LC-MS analyses of serum or plasma in hospitalized patients report a similar sensitivity of ≥90% for the detection of atorvastatin [19]. In a cross-sectional study in patients undergoing cardiac catheterization, results for atorvastatin were found likewise, whereas false negative detects were found in 47% for rosuvastatin which may indicate analytical difficulties with regard to the LC-MS parameters applied in the study [20]. 

The objective measurement of adherence independent of patient reports is undoubtedly a particular advantage of biochemical urine analysis. Nevertheless, it has already been stated by other authors that measuring adherence by biochemical analysis of single spot urine (e.g., by LC-MS) facilitates an insight to a certain time point rather than an overview over a longer period of time [12]. Until now, the relationship between pharmacokinetics and biochemical urine testing for non-adherence to statins has not been further investigated [12]. A pharmacokinetic study with radiolabeled rosuvastatin in six volunteers found total excretion of rosuvastatin from feces and urine as measured by recovery of radioactivity within ten days [21]. Similar data was not retrievable for atorvastatin. In a recent study of 60 patients with and without ASCVD on regular prescription of atorvastatin researchers were able to detect if two or more doses were omitted by LC-MS testing of blood with a sensitivity of 90% [22]. Accordingly, the present results of serial urine testing by LC-MS in volunteers up to one week after the intake of a statin dose may as well help to further narrow down the time window of adherence: One week after the intake atorvastatin was not detected in any of the volunteers’ urine samples and rosuvastatin was not detected in 90%. When applied to patients, a negative result indicates a high likelihood of non-adherence to the prescribed statin during one week or longer prior to collection of the urine sample. Having this information may be helpful to physicians when communicating with patients on non-adherence and possible reasons. However, the present method of biochemical snapshot assessment of adherence cannot distinguish patients with good from those with poor adherence within the prior week. The method is also not able to differentiate between individuals who discontinued the therapy and irregular statin users. These aspects are properties of other measures of adherence, e.g., health claims and medication refill data, or patient reports [23–25]. If it becomes possible at some point to grade quality of adherence to statin therapy based on measuring urine concentrations by LC-MS, factors that have secondary importance in the present study (e.g., dose timing, intermittent use, last-dose effect, and delayed sample collection) will be of greater interest and need further evaluation. In future comparative studies differences and similarities of direct biochemical adherence measurements by LC-MS and established indirect measures such as patient reports (e.g., Medication Adherence Report Scale in German (MARS-D), Rief Adherence Index, etc.) or prescription-based adherence indicators (e.g., proportion of days covered or medication possession ratio) have to be further elucidated [26, 27]. 

In the present study the frequency of non-adherence to statin therapy (13.8%) was in a comparable range to a cross-sectional study utilizing LC-MS analysis of urine in patients with type 2 diabetes (4.5%) but considerably lower than in patients with chronic kidney disease when investigated likewise (34%) [16, 28]. Until now, data from LC-MS analyses of urine for testing adherence to statins in patients with CAD is not retrievable. A cross sectional study in 373 CAD patients testing adherence to atorvastatin by LC-MS analysis of blood plasma detected reduced adherence (as defined by two or more omitted doses) in 8% [29]. Current real-world evidence from a relatively small cohort of 74 German CAD patients enrolled in a general practitioner-led disease management program showed poor adherence to statins in 16.2% measured indirectly by medication possession ratio [30]. A retrospective analysis of a large set of prescription data from the statutory health insurance (approximately 860k patients with dyslipidemia) found high adherence to statin therapy of 84% in patients who were persistent with the therapy. However, at 36 months, only 20.6% of participants persisted with statins [31]. 

Other studies investigating differences in adherence between atorvastatin and rosuvastatin were not found during literature search. The lower proportion of non-adherent patients on prescription of rosuvastatin as observed in the present analysis needs to be further evaluated.

In general, reasons for non-adherence to statin therapy are multi-layered: In a comprehensive umbrella review numerous interacting factors were identified [32]. Among these were non-modifiable sociodemographic factors (e.g., sex, age, ethnicity, education, and socio-economic status) as well as clinical factors (e.g., type of prevention, side effects, statin intensity, and comorbidities) [32–34]. Other factors included healthcare system properties (e.g., healthcare access or co-payments) and modifiable factors of disease knowledge, health literacy, psychological barriers, social support, feelings of self-efficacy, and medical distrust [32–34]. With regards to the present study, some of the factors mentioned above may have contributed to the relatively low proportion of patients with non-adherence in the study population: The population was predominantly male (81.3%), while female sex is known to be associated with non-adherence to statins [35]. Second, mean age (65.9 years) was in a range (50–69 years), where good adherence to statin therapy was found more frequently as compared to other age groups (i.e., < 50 and > 70 years) [36]. In addition, depending on socio-economic status, there exist no or very low co-payments to prescribed medicine, which may have influenced non-adherence in the present study [37]. 

### Limitations

The present study holds several limitations. The sample size of healthy volunteers was rather small with a high proportion of males (81%). Mean age among volunteers differed considerably from CAD patients (37.3 vs. 65.6 years). The limited diversity of the volunteer sample restricts transferability of results to other populations, such as females, older people, or people with comorbidities possibly affecting drug metabolism and excretion. The majority of patients in the CAD cohort were also male. Since women are known exhibit different adherence behavior to statins than men (e.g., due to different tolerability), the present results regarding adherence may not necessarily be transferable to women. A small proportion of included CAD patients had severe renal impairment (five patients with atorvastatin, estimated glomerular filtration rate < 30 mL/min/1.73 m/m^2^, Table 2). Urine excretion of atorvastatin in this small group of patients may differ from that in the rest of the population which may be associated with another period of time after which the intake can be detected.

The cut-off values for non-adherence were set in the range between LOD and LOC/lower calibration limit, which represents a qualitative approach with limited analytic accuracy of measured concentrations. In some urine samples measured rosuvastatin concentrations were above the upper calibration limit, a range with limited analytic accuracy as well, which again does not represent a relevant issue for the present qualitative approach. Furthermore, the LC-MS analyses of volunteer urines were performed after a single dose of a high-potency statin. Statin therapy usually reflects a daily intake over a longer time period with possible differences in excretion kinetics as compared to a single dose intake which limits translational validity of the cut-off values.

## Conclusion

LC-MS analysis of urine is a feasible method for direct testing of non-adherence during one week or longer to high-potency statins in ambulatory patients with CAD. The findings currently obtained in a predominantly male, older population cannot necessarily be transferred to other patient groups. The frequency of non-adherence to statin therapy among German ambulatory CAD patients was comparable to other studies of direct measures. Further evaluation of associated factors of non-adherence directly measured by biochemical methods and its correlations with other measures such as patient reports should be subject of future investigations.

## Supplementary Information

Supplementary Material 1.

## Data Availability

All data relevant to this study are included in the article or uploaded as supplementary information.

## Abbreviations

ASCVD: Atherosclerotic cardiovascular disease
CAD: Coronary artery disease
DIN: German Institute for Standardization
LC-MS: Liquid chromatography/mass spectrometry
LDL-C: Low-density lipoprotein cholesterol
LOD: Limit of detection
LOQ: Lower limit of quantification
UP: Ultra performance
