# Supplementary material for: Biochemical urine analysis of atorvastatin and rosuvastatin by LC-MS: a pilot study of an objective method to assess non-adherence
Source: BMC Cardiovasc Disord. 2026 Jan 9;26:83. doi: 10.1186/s12872-025-05475-0 (PMC12836941; doi:10.1186/s12872-025-05475-0)
Supplement: Supplementary file 1 — Supplementary Material 1. [file 12872_2025_5475_MOESM1_ESM.pdf]

Supplementary Table 1 – Volunteer urine concentrations

a) Study drug: 40 mg Atorvastatin					b) Study drug: 20 mg Rosuvastatin				
**Study ID**	**Urine concentration (ng/mL)**				**Study ID**	**Urine concentration (ng/mL)**			
	Day 1	Day 2	Day 3	Day 7		Day 1	Day 2	Day 3	Day 7
A1	<1.0*	<1.0*	<1.0*	<1.0*	R1	308	94	7.0	0.0*
A2	44	2.0	<1.0*	<1.0*	R2	103	25	8.9	0.5*
A3	73	31	2.9	<1.0*	R3	43	31	7.3	0.6*
A4	9.0	5.0	<1.0*	<1.0*	R4	0.2*	0.4*	0.2*	0.3*
A5	66	4.0	<1.0*	<1.0*	R5	59	23	12	0.3*
A6	22	4.3	1.4	1.3*	R6	188	60	46	2.6
A7	24	2.3	1.7	1.3*	R7	259	42	4.1	0.5*
A8	7.0	2.9	1.7	1.2*	R8	196	38	18	0.4*
A9	19	4.6	1.5	1.3*	R9	diseased excluded			
A10	23	9.3	1.5	1.3*	R10	123	74	21	0.5*
A11	5.1	1.8	1.3*	1.2*	R11	503	87	26	0.8*
A12	119	4.6	2.0	1.1*					
A13	21	4.9	2.0	1.2*					

Supplementary Table 1: Concentrations (ng/mL) of high-potency statins of **a)** atorvastatin and **b)** rosuvastatin in volunteer urine samples measured by liquid chromatography/mass spectrometry (LC-MS). Urine samples were taken one, two, three, and seven days after the oral intake of a single dose of 40 mg atorvastatin or 20 mg rosuvastatin. An ACQUITY ultra performance (UP) LC system (Waters, Milford, USA) with an ACQUITY TQD quadruple tandem mass spectrometer with electrospray ionization and column oven (MS/MS, Waters, Milford, USA) was used. Due to illness one subject was excluded, because adequate storage of urine samples could not be warranted. The limits of detection LOD (LODs) of atorvastatin and rosuvastatin were 1.37 ng/mL and 0.93 ng/mL as determined on spiked urine samples. The cut-off values for non-adherence derived from LC-MS analyses were defined qualitatively based on LODs. For atorvastatin cut-off was set <1.40 ng/mL, for rosuvastatin <1.00 ng/mL.
* indicates a value below the cut-off value for detection of statin.

Supplementary Figure 1 – Exemplary chromatograms: Volunteers' urine samples

a) Day 1

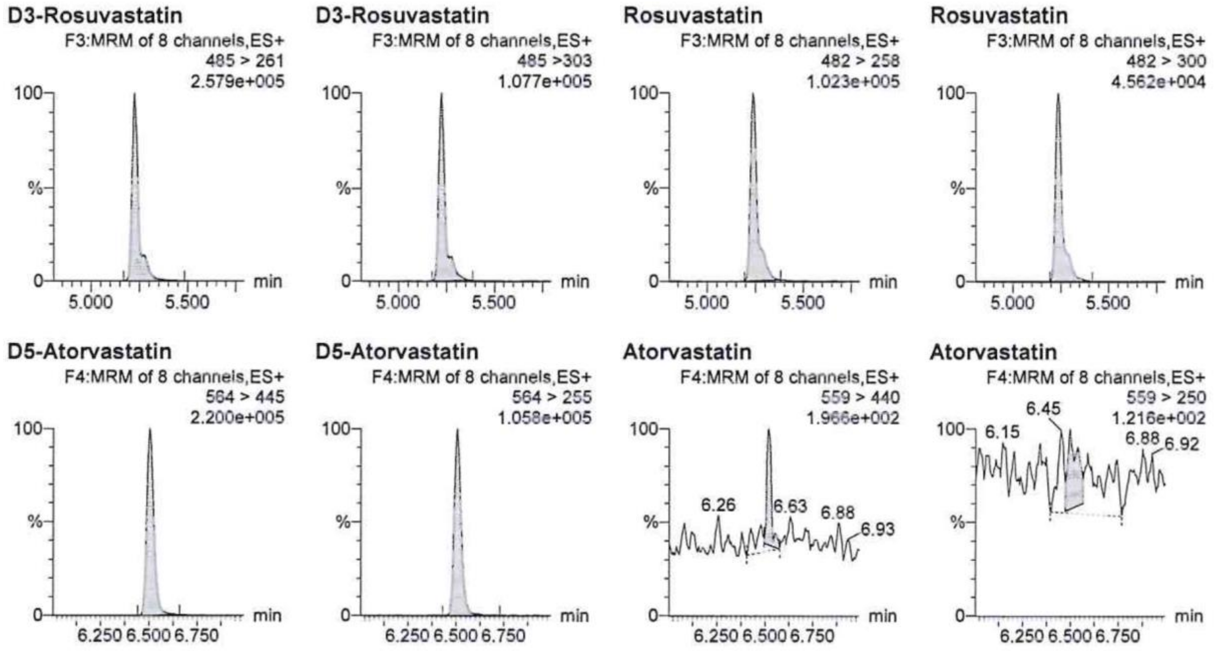

b) Day 7

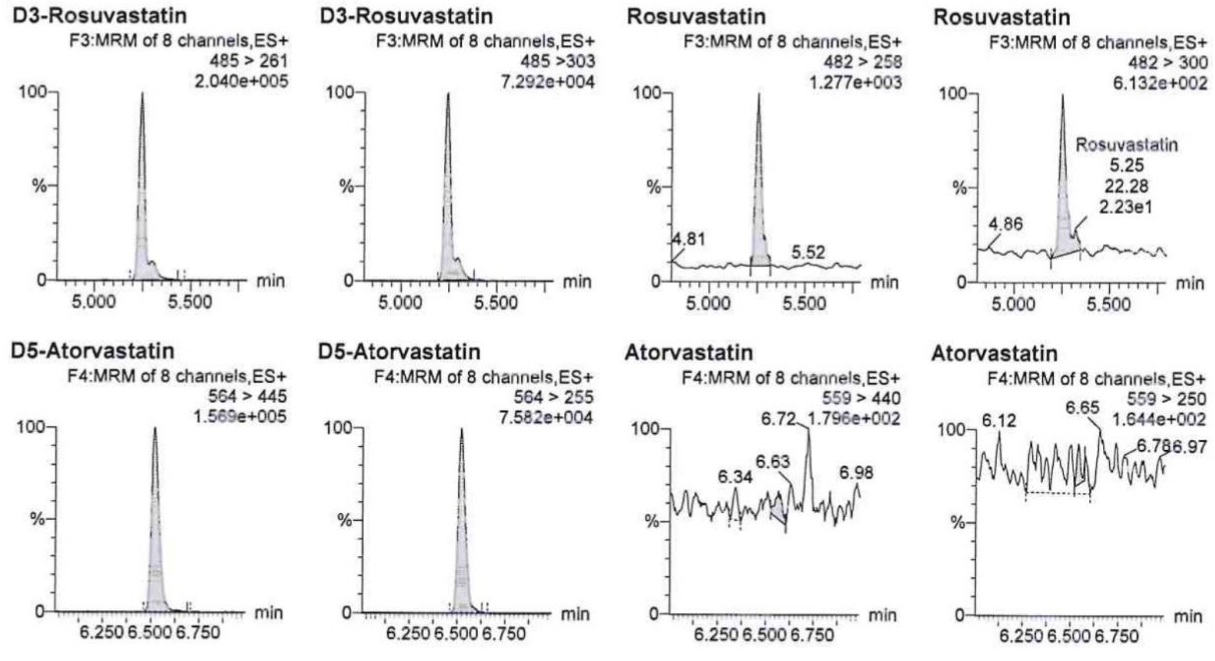

Supplementary Figure 1: Exemplary chromatograms of liquid chromatography/mass spectrometry (LC-MS) analysis of volunteer urine samples **a)** one day and **b)** seven days after the intake of 40 mg atorvastatin. The graphs show the relative response (%) as a function of the retention time (min), in the upper row for deuterated (D3)-rosuvastatin and the test substance and in the bottom row for deuterated (D5)-atorvastatin and the respective test substance for two different transitions. a) and b) indicate a positive result for the detection of rosuvastatin and a negative for atorvastatin. An ACQUITY ultra performance (UP) LC system (Waters, Milford, USA) with an ACQUITY TQD quadruple tandem mass spectrometer with electrospray ionization and column oven (MS/MS, Waters, Milford, USA) was used.
